# Training loads and practices of competitive organ-recipients at the British and World Transplant Games

**DOI:** 10.3389/fspor.2024.1445491

**Published:** 2024-11-29

**Authors:** Thomas Hames, Sheila Leddington-Wright, C. Douglas Thake, Stefan De Smet, Mike Price

**Affiliations:** ^1^ School of Health and Life Sciences, Faculty of Engineering, Environment and Science, Coventry University, Coventry, United Kingdom; ^2^ Physical Activity, Sport and Exercise Sciences, Institute of Health and Wellbeing, Coventry University, Coventry, United Kingdom; ^3^ Exercise Physiology Research Group, Department of Movement Sciences, KU, Leuven, Belgium

**Keywords:** solid-organ transplant, session RPE, training intensity, training frequency, training duration, training load, resistance training, aerobic training

## Abstract

**Background:**

Little information is available regarding the TL and training practices of competitive athletes who are solid-organ recipients. This study characterized TL and practices of competitive organ-recipients at the British and World Transplant Games, 2017.

**Methods:**

Questionnaire data was gathered from 220 participants regarding sporting events and type, frequency, duration and intensity of training sessions undertaken. TL for each training session (session duration × session rating of perceived exertion [RPE]) and total TL were determined.

**Results:**

Overall participants trained 5 ± 3 times per week at an RPE of 5 ± 2 AU for between 60 and 75 min per session (TL ∼1,500–1,875 AU). Most participants (*n* = 176; 79.7%) reported training three or more times each week. Approximately half (49%) the participants reported undertaking resistance training each week (3 ± 2 sessions per week, RPE of 6 ± 2 AU, 45–60 min per session; TL ∼810–1,080 AU, respectively). Of those participants undertaking resistance training 75% undertook two or more sessions each week. Participants generally undertook most events within a predominant sport with one or two events in a secondary sport. Typical weekly TL for the whole group was 2,762 ± 3,583 AU with considerable variation within and across sports (cycling 4,383 ± 4,005 AU; field athletics 3,671 ± 6,181 AU; court-based sports 2,645 ± 3,308 AU; high physical demand sports [e.g., skiing, triathlon] 2,595 + 2,247 AU; track athletics 2,547 ± 2,664 AU; swimming 2,091 ± 1,070 AU; low physical demand sports [e.g., darts, petanque, walking] 1,182 + 801 AU). Sports-specific TL for predominant and secondary sports was ∼1,500 AU. Resistance training and gym-based aerobic training were the most frequent non-specific training components undertaken. Most competitors (79%) were active in sport prior to transplant.

**Conclusion:**

The wide range of TL and components undertaken by athletes with solid-organ transplants likely reflects the multiple and diverse event participation of competitive organ-recipients as well as the diverse physical fitness profiles and incentives to participate. Optimization of TL both for multiple competitive sports and maintenance of health should be considered for these athletes.

## 1 Introduction

The fundamental aim of sport participation following organ transplant is the promotion of a healthy lifestyle whilst simultaneously increasing public awareness of organ donation (1). To showcase the physiological abilities of transplant recipients the World Transplant Games was developed. The first Games in 1978 involved 99 competitors increasing to 1,500 in 2017, with an anticipated 2,500 competitors in 2023 (2, 3). With such a rise in participation guidance on training activities is warranted, particularly as regular exercise is known to negate various side effects and functional impairments that result from transplant surgery and/or prescribed medications, such as; decreased bone mineral density, muscle weakness, hypertension, hyperlipidaemia, hypercholesterolemia, metabolic syndrome, impaired glucose tolerance, reduced aerobic capacity and exercise tolerance (4–9). However, despite the known benefits of exercise on transplant recipients there are also potential downsides of strenuous physical activity in this population (10). Furthermore, information regarding training characteristics (i.e., intensity, frequency, duration), training activities and guidance to support those transplant recipients undertaking athletic development is sparse (11). As competitors at national and World Transplant Games are eligible to compete in up to five different events (3), determining and understanding training load (TL) is important in understanding a healthy adaptation to training (12).

Although some transplant recipients may regain near full pre-transplant athletic potential (13), approximately 23% of competitors taking part in the USA Transplant Games were deemed as inactive or undertaking less physical activity than recommended by conventional guidelines at that time (14) [i.e., ‘Active’ being considered as at least three sessions of aerobic exercise for 30 min, at or above a rating of perceived exertion (RPE) of 13 each week (15)]. Furthermore, the majority (51%) of competitors at the Latin American Transplant Games only started training in the 12 months before the Games (4). Within these studies, ‘training’ was summarised only as cardiovascular and strength training (14) or represented a range of exercise modes culminating in ∼7 h training each week (4). A recent position stand (9) reported that exercise training pre-transplant was safe, but there was insufficient evidence to provide specific guidelines on the training characteristics. In addition, to obtain benefits, exercise training should be of moderate to vigorous-intensity level, 3–5 times a week for a minimum of 8 weeks. Despite this general insight into pre-transplant exercise training and the training habits of competitive organ-recipients knowledge is lacking in relation to sport specific TL and event preparation. Although consensus statements regarding TL exist for non-transplant athletes [e.g., (16, 17)], and exercise recommendations are available for organ-recipients (9) no specific training guidelines are available for competitors who are recipients of solid-organ transplants. When considering most athletes with solid organ transplants regularly take immunosuppressant medication (11), understanding TL and the potential for disrupting the delicate balance between training adaptations and health is even more important for this population. Therefore, the aim of this study was to determine the TL and practices of competitive organ-recipients. It was hypothesized that due to the likely range of competitive events available to this population event-specific TL may represent only a small proportion of total TL.

## 2 Method

### 2.1 Participants

Following University ethical approval (Ref: P52535) and informed consent, a survey-based study was completed by 220 (Male; 139, female; 81) English speaking competitive organ-recipients. Gatekeeper consent for National and International athletes was provided through Transplant Sport UK and national team managers and the board of World Transplant Games Federation, respectively. Inclusion criteria was successful entry into the 2017 British or World Transplant Games (BTG; WTG) thus confirming athletes had met the required BTG/WTG conditions for competition (i.e., having received one or more life supporting allografts (e.g., kidney, liver, lung, heart, stem cell), be a minimum of 6 months post-transplant with a stable allograft and being medically fit as signed off by their doctor (3).

### 2.2 Survey procedure

The survey was made available using the Bristol Online Survey platform and advertised through the World Transplant Games Federation and consenting national team web pages. Participants completed the survey using the same web link on these sites which included built in participant consent. Further recruitment to web adverts occurred through word of mouth at both the BTG (Glasgow; 27th–30th July 2017) and the WTG (Malaga; 24th–30th June 2017) where tablets and hard copies of the questionnaire were available to suit the participants’ preference of completion. To assess clarity and practicality of survey completion, the survey was piloted by four competitive organ-recipients. The pilot group reported the time demand to complete the survey was acceptable (15–20 min) and questions facilitated reflection of participants transplant and training journey as well as their achievements. The questionnaire (Supplementary Material) consisted of a total of 63 questions relating to each competitors’ personal and transplant characteristics, training practices pre- and post-transplant, recovery from exercise and training advice and support experienced. Participant consent was built into the first section of the questionnaire along with details of transplant type, age, mass (kg), height (m). Body mass index (BMI) was subsequently calculated as body mass/height squared (14). Those questions specifically related to training practices (i.e., the number and type of sporting disciplines competed in, the number, type and intensity of training undertaken) are reported here. Questions relating to TxA characteristics (sex, age, height, body mass index, nationality and ethnicity), transplant history (initial reason for transplant, age at transplant), medications, complications, source of and training advice and reasons for attending Tx Games have been reported previously (11) but briefly considered here for context.

As competitive organ-recipients compete in multiple sports and events within them, participants were required to indicate which sport was their primary competitive focus. Sports included those typically available at transplant events; track athletics, field athletics, cycling, swimming and court-based events, as well as high physiological demand sports (HPD; i.e., triathlon, skiing etc.) and low physiological demand sports (LPD; i.e., darts, petanque, walking etc.). Participants were asked to report the type of training usually undertaken during a typical week in season and thus corresponding to the time of completion of the questionnaire (12th June 2017 to 1st September 2017) along with both the duration and intensity of each session (18, 19). Session intensity was estimated based upon Borgs 1–10 rating of perceived exertion scale (RPE) (20) due to its ease of use. A full explanation of the scale was provided within the questionnaire. Training session duration was reported as 15 min blocks. While this duration does not reflect shorter session details it allows control over recall error and a reasonable estimation of session duration (21). Training session modality options comprised of; gym-based sessions (resistance training, aerobic training, exercise classes), athletic track (sprint, middle and long distance), athletic field (jump and throwing), cycling (sprint, middle and long distance), swimming (sprint, middle and long distance), court-based sports (volleyball, badminton, basketball, tennis) and “other”. Individual TL were quantified as per methods of Alexiou and Coutts (22) (mean session intensity = RPE × duration; arbitrary units, AU) for each training modality. Total weekly TL was then calculated as the sum of all training modality TL undertaken.

### 2.3 Statistical analyses

Data was initially checked for normality using the Shapiro-Wilkes test with summary data reported as mean ± standard deviation (SD). Data was analysed using SPSS (v26, IBM, Chicago). Differences between total TL of each sporting discipline, the number of sports undertaken and the number of events in each competitor's main sport were analysed by independent groups one-way analysis of variance (ANOVA). The frequency of each training type (resistance, aerobic-gym based, track, field, cycling, swimming, court-based) in relation to each sport were expressed as frequency counts and as the percentage of athletes in each sport. The frequency, duration and intensity of training for the whole group were described as mean ± SD and expressed as frequency counts in relation to their various categories (frequency; 1, 2, 3 etc. sessions per week, duration; up to 15 min, 16 min up to 30 min etc. per session, intensity; low = RPE 1–4, moderate = RPE 5–7, high = RPE 8–10). Training load for those participants undertaking and not undertaking resistance training was compared using an independent *t*-test, as were total training load, frequency, duration and intensity of training for those participants reporting competing at BTG and WTG standard. The distribution of TL (500 AU category increments) was also determined. When considering involvement in sport and physical activity prior to transplant, categories of “not undertaken sport or had only done so at school”, participating in sport at a “recreational or club standard” and participating at “county or national standard” were used. Due to the low frequency of some training components reported by competitors no meaningful statistical analysis could be undertaken, accordingly the magnitude of each predominant component across sports is described. Effect sizes (ES) for *t*-tests were interpreted as; small (<0.2), moderate (>0.5), and large (>0.8) whereas ES from ANOVA analysis (Partial eta squared; ƞp^2^) were interpreted as; small effect (<0.01), medium (>0.06) and large effect (>0.14) (23). Where SPSS returned *P* values of 0.000 these are reported as *P* < 0.001.

## 3 Results

### 3.1 Participant characteristics

The general characteristics of participants grouped according to predominant sporting discipline are shown in Table 1. Overall, 63% or competitors were male and 37% were female, which was consistent across most sporting disciplines. Competitors age for males and females was similar [F_(1,215)_ = 0.172, *P* = 0.679, ƞp^2^ = 0.001] with male participants being heavier than females [F_(1,213)_ = 51.169, *P* = 0.001, ƞp^2^ = 0.094], but with similar BMI [F_(1,211)_ = 5.951, *P* = 0.016, ƞp^2^ = 0.027]. The specific distribution of organ transplant type per sport is provided as (Supplementary Table S1).

**Table 1:** Characteristics of athletes with solid organ transplant grouped according to predominant sport, as per British Transplant Games and World Transplant Games competitions.

Transplant competitors by main sporting event			All	Track	Field	Cycling	Swimming	Court-based sports	High physical demand	Low physical demand
Sex	All		218	51	30	16	26	70	6	19
	Male		138	32	19	11	13	45	2	16
	Female		80	19	11	5	13	25	4	3
Age	All	Mean ± SD	45 ± 15	46 ± 16	43 ± 13	48 ± 11	46 ± 19	42 ± 15	36 ± 14	49 ± 13
		Range	77	60	53	39	65	63	37	36
	Male	Mean ± SD	45 ± 16	47 ± 17	43 ± 15	48 ± 11	50 ± 21	41 ± 15	35 ± 21	48 ± 13
		Range	77	60	53	35	65	63	29	36
	Female	Mean ± SD	44 ± 14	46 ± 13	42 ± 9	49 ± 14	43 ± 16	43 ± 15	37 ± 14	56 ± 14
		Range	53	45	27	39	49	50	32	25
Mass (kg)	All	Mean ± SD	73 ± 16	67 ± 10	85 ± 18	68 ± 10	69 ± 15	75 ± 20	65 ± 10	82 ± 14
		Range	103	42	67	40	59	124	26	46
	Male*	Mean ± SD	78 ± 15	70 ± 9	89 ± 17	72 ± 8	78 ± 13	79 ± 17	71 ± 14	85 ± 13
		Range	97	38	56	29	46	96	20	40
	Female	Mean ± SD	64 ± 12	61 ± 10	77 ± 18	59 ± 10	60 ± 11	68 ± 24	61 ± 6	63 ± 1
		Range	63	42	59	24	36	124	15	2
Height (cm)	All	Mean ± SD	172 ± 11	171 ± 10	176 ± 10	173 ± 11	172 ± 11	172 ± 12	170 ± 8	172 ± 8
		Range	54	49	44	39	41	64	20	25
	Male	Mean ± SD	177 ± 9	174 ± 10	181 ± 8	178 ± 6	179 ± 11	177 ± 9	176 ± 6	175 ± 6
		Range	55	49	24	19	36	43	8	18
	Female	Mean ± SD	164 ± 8	166 ± 10	166 ± 8	162 ± 5	165 ± 5	163 ± 9	167 ± 6	160 ± 2
		Range	35	34	25	13	19	38	15	4
BMI	All	Mean ± SD	24.5 ± 4.1	22.8 ± 3.3	27.4 ± 4.6	22.6 ± 1.9	23.3 ± 3.6	25.3 ± 5.5	22.3 ± 1.9	27.9 ± 3.7
		Range	25	16	17	7	15	40	5	13
	Male	Mean ± SD	24.9 ± 3.9	23.3 ± 3.5	27.1 ± 4.0	22.8 ± 1.6	24.4 ± 3.4	25.3 ± 4.2	22.8 ± 3.1	28.3 ± 3.8
		Range	23	16	15	7	13	23	4	13
	Female	Mean ± SD	23.7 ± 4.0	21.9 ± 3.0	28.0 ± 5.9	22.4 ± 2.6	22.3 ± 3.6	25.5 ± 7.3	22.1 ± 1.6	24.7 ± 1.3
		Range	19	10	16	7	12	40	4	2

(*n* = 218/220, two competitors did not identify with a main sport). *Represents a significant difference between males and females (*P* < 0.05).

### 3.2 Sport and event participation

**Figure 1:**
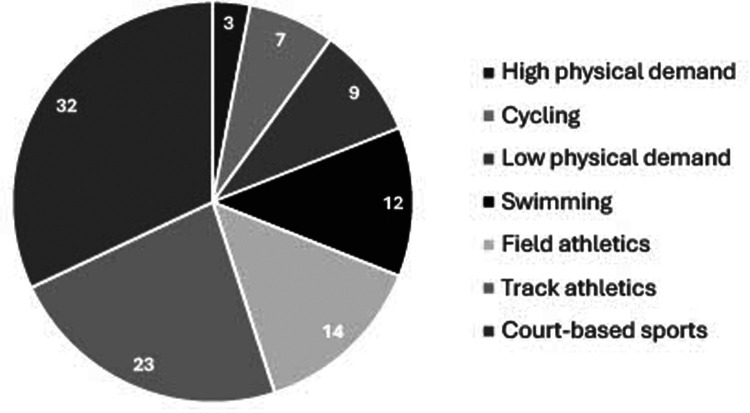
Predominant competitive sports reported across all competitors. Values shown are percentages (%). Legend represented clockwise from High physical demand sports (3%) to Court-based sports (32%).

When considering involvement in sport and physical activity prior to transplant, 29% (*n* = 63) of competitors had either not undertaken sport or had only done so at school, with 79% of these (*n* = 49/63) having never engaged with sport at any level. Those participating in sport at a recreational or club standard pre-transplant represented 48% of competitors with 23% having competed at county or national standard. The most predominant sports represented across the group were court-based sports, track athletics, field athletics and swimming (Figure 1). There was a difference in the number of different sports undertaken across each predominant sporting group [F_(6,211)_ = 2.996, *P* = 0.008, ƞp^2^ = 0.079] (Table 2), whereby LPD competitors participated across more sports compared to other competitors except for court based competitors (LPD vs. track *P* = 0.005; field *P* = 0.030; swimmers *P* < 0.010; cyclists *P* = 0.020, HPD *P* = 0.026). Swimmers also undertook less sports compared to court-based competitors (*P* = 0.005). The total number of events competed in per sport also differed across sporting groups [F_(6,211)_ = 2.316, *P* = 0.035, ƞp^2^ = 0.062] with swimmers undertaking the greatest number of events (5 ± 3) compared to most other sports (swimmers vs. track *P* = 0.011; field *P* = 0.006; cycling *P* = 0.004; court-based sports *P* = 0.001 and LPD *P* = 0.042). When considering events within their primary sport, track and swim competitors undertook more sport specific events (3 ± 2 and 4 ± 2, respectively) than field, cyclists, court, HPD and LPD competitors [∼2 events; F_(6,211)_ = 11.591, *P* = 0.000, ƞp^2^ = 0.248] (Table 2).

**Table 2:** Sporting event participation of competitive organ-recipients at the British and World Transplant Games.

Sport	Current sporting event participation				
	Number of athletes (%)	Total sporting disciplines (*n*)	Total events (*n*)	Specific events (*n*)	Non-specific events (*n*)
All sports	218 (100%)	2 ± 1	4 ± 2	2 ± 2	1 ± 2
Male	138 (63.3%)	2 ± 1	4 ± 2	2 ± 2	2 ± 2
Female	80 (36.7%)	2 ± 1	4 ± 2	2 ± 2	1 ± 2
Track athletics	51 (23%)	2 ± 1	4 ± 2	3 ± 2	1 ± 1
Male	32 (63%)	2 ± 1	4 ± 2	3 ± 2	1 ± 1
Female	19 (37%)	2 ± 1	4 ± 2	3 ± 1	1 ± 2
Field athletics	30 (14%)	2 ± 1	4 ± 1	2 ± 1	2 ± 1
Male	19 (63%)	2 ± 1	4 ± 2	2 ± 1	2 ± 2
Female	11 (37%)	2 ± 1	3 ± 3	2 ± 1	2 ± 2
Cycling	16 (7%)	2 ± 1	3 ± 1	2 ± 1	1 ± 1
Male	11 (69%)	2 ± 1	3 ± 1	2 ± 1	1 ± 1
Female	5 (31%)	2 ± 0	3 ± 2	2 ± 1	2 ± 1
Swimming	26 (12%)	1 ± 1	5 ± 3	4 ± 2	1 ± 2
Male	13 (50%)	1 ± 1	6 ± 4	5 ± 3	1 ± 3
Female	13 (50%)	1 ± 1	4 ± 2	3 ± 2	1 ± 1
Court-based sports	70 (32%)	2 ± 1	3 ± 2	2 ± 1	1 ± 2
Male	45 (64%)	2 ± 1	3 ± 2	2 ± 1	2 ± 2
Female	25 (36%)	2 ± 1	3 ± 2	2 ± 1	1 ± 1
High physical demand sports	6 (3%)	2 ± 1	4 ± 2	2 ± 2	2 ± 2
Male	2 (33%)	2 ± 0	5 ± 0	2 ± 1	4 ± 1
Female	4 (67%)	1 ± 1	3 ± 3	3 ± 2	1 ± 1
Low physical demand sports	19 (9%)	3 ± 1	4 ± 3	1 ± 1	2 ± 2
Male	16 (84%)	2 ± 1	3 ± 3	1 ± 1	2 ± 2
Female	3 (16%)	3 ± 1	4 ± 2	2 ± 1	2 ± 2

Number of athletes refers to out of whole group/sporting discipline as indicated.

### 3.3 Training frequency, intensity and duration

**Figure 2:**
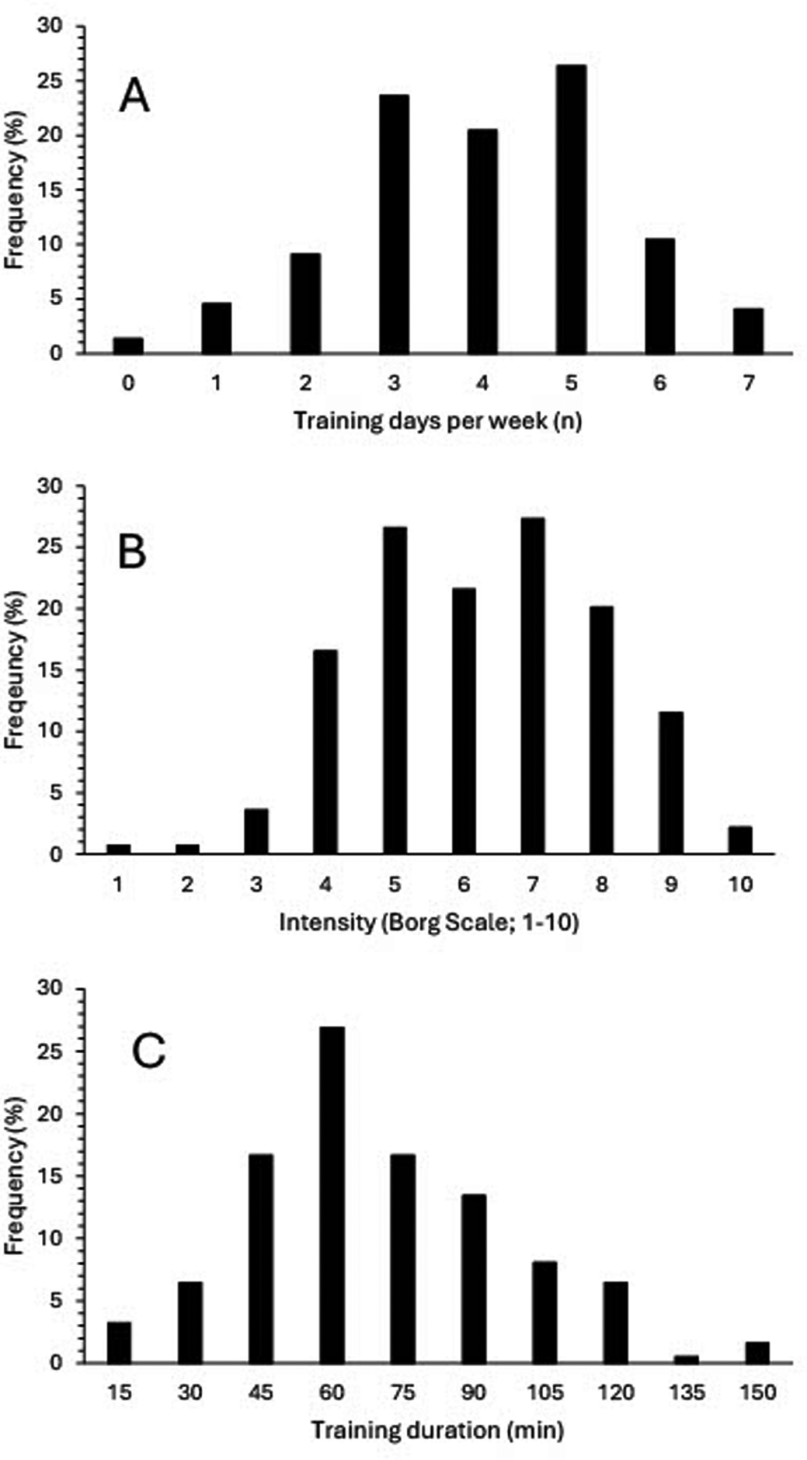
Distribution of training frequency as indicated training days per week **(A)**, training intensity as indicated by RPE (0–10 Borg scale) **(B)** and training duration expressed in 15 min blocks **(C)** across all training sessions for all competitors.

Across the whole group, participants undertook training 5 ± 3 times per week at an intensity of 5 ± 2 AU for between 60 and 75 min per session (representing TL of ∼1,500–1,875 AU). The distributions of training frequency, intensity and duration are shown in Figures 2A–C. Most participants (*n* = 176; 79.7%) reported training three or more times each week. Of the 20.3% of participants reporting training less than three times each week, only 2.7% (*n* = 6) reported no specific training being undertaken. For 10 of the 16 training modalities intensity was rated as 4 (range 3–8) but with sport specific training (i.e., cycling in cyclists, swimming in swimmers etc.) rated as 5 or above (Supplementary Table S2). Where sport specific training was categorised as sprint, middle- and long-distance sessions (i.e., running, swimming and cycling), RPE approximated 7, 6 and 5, respectively. Exercise session durations of 30–45, 45–60 and 60–75 min were reported in 18.2%, 24.6%, 15.5% of participants. Subsequently, 89.3%, 71.1% and 47% of participants exercised for over 30, 45 and 60 min per session, respectively. The individual components of training load (i.e., frequency, RPE, duration) for each training modality according to predominant sport are shown in Supplementary Table S2.

#### 3.3.1 Resistance training

Resistance training was reported by 49% (*n* = 108) of participants, with a mean frequency of 3 ± 2 sessions per week at an intensity of 6 ± 2 AU for between 45 and 60 min (representing TL of 810–1,080 AU, respectively; mean ± SD, 1,108 ± 1,764 AU). Of those participants undertaking resistance training 75% undertook two or more sessions each week. For those participants undertaking resistance training the remainder of the weekly training load represented 2,690 ± 3,362 AU, which was greater than compared to that of participants who did not report undertaking resistance training (1,741 ± 1,872 AU; *P* = 0.011).

### 3.4 Total training load

**Figure 3:**
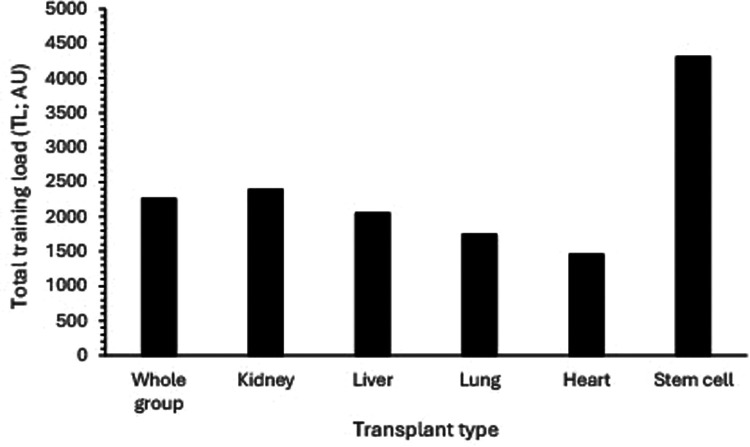
Total training load (TL) in relation to transplant type. (NB: Standard deviations omitted for clarity; Whole group sd = 3,411 AU, Kidney = 3,095 AU, Liver = 2,979 AU, Lung = 1,457 AU, Heart = 1,453 AU, Stem cell = 7,003 AU).

The mean TL of competitors who indicated they trained specifically for their events (*n* = 180) was 2,762 ± 3,583 AU. No differences in training load were observed for competitors across sporting groups [F_(6,173)_ = 1.272, *P* = 0.273, ƞp^2^ = 0.042], though, based on standard deviations, variation was considerable. Where competitors who indicated they did not train specifically for their main event (*n* = 38/218 competitors) were included, the mean training load was 2,260 ± 3,411 AU, again with no difference between groups observed [F_(6,211)_ = 2.015, *P* = 0.065, ƞp^2^ = 0.054]. Although no differences in TL were observed in relation to transplant organ [F_(4,169)_ = 1.982, *P* = 0.099, ƞp^2^ = 0.045], the resultant effect sizes suggested meaningfully greater TL in stem cell recipients when compared to all other transplant types (ƞp^2^ = 0.379–0.675); for kidney recipients when compared to liver, lung and heart recipients (ƞp^2^ = 0.112–0.403); for liver recipients when compared to heart and lung recipients (ƞp^2^ = 0.268, 0,138, respectively) and for heart recipients when compared to lung recipients (ƞp^2^ = 0.195) (Figure 3).

#### 3.4.1 Competition standard

**Figure 4:**
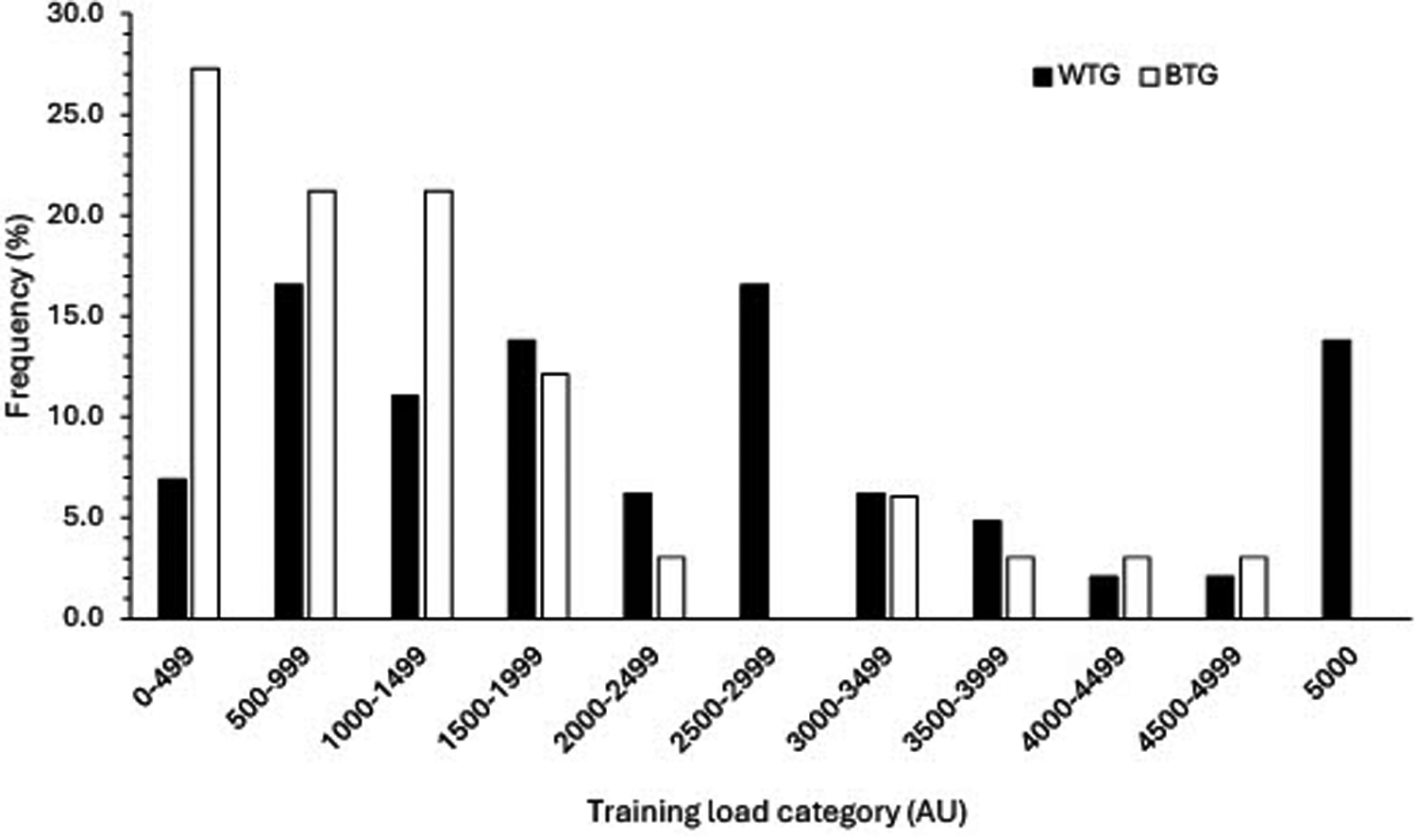
Distribution of training load between participants competing predominantly at the British and World Transplant Games.

Total TL was greater for those participants predominantly competing at the WTG (*n* = 143; 81%) than for those at the BTG (3,088 ± 2,882 vs. 1,376 ± 1,202 AU, respectively; ES = 0.482, *P* = 0.006). However, most participants undertook more than two sessions each week (81%, 75%, respectively). For those competing predominantly at the BTG the most common TL category was 0–499 AU (27.2%, 6.9% for BTG and WTG, respectively) (Figure 4). Thereafter the distribution per category for BTG competitors decreased. For those competing at WTG standard, there was a more uniform distribution of total TL between 500 and 3,000 AU and a further peak at >5,000 AU (13.8%). Although there were no differences in the duration of training sessions between BTG and WTG competitors (up to 60 min, ES = 0.213, *P* = 0.117), training frequency (4.2 vs. 3.5 sessions per week, respectively, ES = 0.466, *P* = 0.002) and training intensity (5.4 vs. 4.7 AU, respectively, ES = 0.355, *P* = 0.025) were greater for those competing at the WTG. A greater proportion of participants competing at the WTG undertook resistance training than for those competing at the BTG (52, 42%, respectively). However, the distribution of resistance training sessions per week was similar for both groups.

### 3.5 Training modalities

Table 3 shows the frequency of the different training modalities undertaken in relation to each sporting group. Resistance training was the most common training mode reported across all participants (*n* = 93; 43%). Court-based sports (*n* = 67; 31%) and gym based aerobic training (*n* = 65, 30%) were the second most reported training types across all competitors. Where gym based aerobic training demonstrated a similar distribution across sports to resistance training, court-based sports were predominantly undertaken by court sport players (i.e., *n* = 51/73; 71% of court-based athletes). The remaining sport specific activity modes were predominantly undertaken by those athletes identifying themselves with these modes as their main sporting discipline (i.e., swimming in swimmers). With the exception of track athletics (57%), sport specific training modes were undertaken by 70%–88% of competitors at ∼1,500 AU with no differences observed across predominant sporting disciplines [F_(4,128)_ = 0.905, *P* = 0.463, ƞp^2^ = 0.027] (Table 4). Cyclists, however, appeared to undertake a greater specific TL than other sports (2,792 ± 2,290 UA) although ES was low (ES = 0.027). Training loads of similar magnitude to specific training (i.e., ∼1,500 AU) were also observed for non-specific modes.

**Table 3:** Frequency of training modalities in relation to predominant sport.

Training modality	Sporting event							
	Total *n* = 218	Track *n* = 51	Field *n* = 30	Cycling *n* = 16	Swim *n* = 26	Court *n* = 70	HPD *n* = 6	LPD *n* = 19
	*n* (%)	*n* (%)	*n* (%)	*n* (%)	*n* (%)	*n* (%)	*n* (%)	*n* (%)
Gym resistance	93 (43)	24 (47)	21 (70)	7 (44)	9 (35)	25 (36)	2 (33)	5 (26)
Gym aerobic	65 (30)	15 (29)	14 (47)	5 (31)	4 (15)	21 (30)	4 (66)	2 (11)
Gym classes	28 (13)	5 (10)	3 (10)	3 (19)	1 (4)	6 (9)	–	–
Track/running	53 (24)	29 (57)	8 (27)	2 (13)	2 (8)	8 (11)	3 (50)	1 (5)
Field athletics	31 (14)	3 (6)	21 (70)	–	1 (4)	3 (4)	2 (33)	1 (5)
Cycling	30 (14)	6 (12)	–	14 (88)	2 (8)	6 (9)	3 (50)	1 (5)
Swimming	40 (18)	6 (12)	4 (13)	2 (13)	19 (73)	4 (6)	5 (83)	-
Court-based	67 (31)	1 (2)	4 (13)	3 (19)	2 (8)	51 (73)	1 (17)	6 (32)
Did not train for specific event	38 (17)	9 (18)	3 (10)	2 (13)	6 (12)	9 (13)	–	9 (47)

Total values represent those competitors reporting each training component undertaken (*n*) and as a percentage of all competitors in each sport. Swim, swimming; Court, court based sports; HPD, high physical demand; LPD, low physical demand.

**Table 4:** Total training load and training load for each training modality grouped by predominant sport.

Training modality	Sport						
	Track	Field	Cycling	Swim	Court	HPD	LPD
Gym resistance	822 ± 986	1,904 ± 3,190	999 ± 924	687 ± 556	1,015 ± 1,116	623 ± 711	564 ± 407
Gym aerobic	1,151 ± 1,024	927 ± 898	852 ± 856	960 ± 525	814 ± 642	510 ± 369	390 ± 297
Gym classes	978 ± 1,280	540 ± 393	1,345 ± 474	90^c^	578 ± 423	–	–
Track	1,525 ± 1,496	724 ± 674	450 ± 212	1,268 ± 435	767 ± 581	770 ± 735	480^c^
Field	295 ± 153	1,561 ± 3,391	–	45^c^	2,025 ± 2,924	1,575 ± 1,846	420^c^
Cycling	1,328 ± 1,350	–	2,795 ± 2,290	225 ± 21	713 ± 322	380 ± 346	720^c^
Swimming	305 ± 225	941 ± 704	1,388 ± 689	1,442 ± 869	439 ± 325	489 ± 429	–
Court-based	1,500^c^	150 ± 77	470 ± 321	390 + 212	1,863 ± 2,584	90^c^	630 ± 784
Total training load 1^a^	2,098 ± 2,604	3,304 ± 5,958	3,835 ± 4,018	1,608 ± 1,295	2,305 ± 3,212	2,595 ± 2,247	622 ± 830
Total training load 2^b^	2,547 ± 2,664	3,671 ± 6,180	4,383 ± 4,005	2,091 ± 1,070	2,645 ± 3,309	2,595 ± 2,247	1,182 ± 801

Note that values reflect mean values for those participants who reported undertaking each training component, and not the total number of participants in each sport. (NB: see Table 3 for *n* of each).

^a^ Total training load 1: Mean training load of all competitors according to main sport, including those who do not actively train (*n* = 218).

^b^ Total training load 2: Mean training load only inclusive of the competitors who do train for their sport (*n* = 180).

^c^ *n* = 1 competitor.

## 4 Discussion

This is the first study to report event participation, training practices and training load of organ recipients competing at the British and World Transplant Games. Our study, comprising over 200 transplant sport athletes, represents the most comprehensive analysis of transplant athletes to date and, as such, presents new understanding central to the future development of training prescription for such athletes. The study demonstrated that 71% of competitors were active in sport prior to their transplant with Games participants competing in multiple events within their primary sport and, generally, at least one secondary sport. Multiple and diverse event participation as well as the diverse physical fitness profiles and incentives to participate likely explain the resultant wide range of training modalities and training loads of the competitors observed. The most frequent training modalities were resistance training and gym-based aerobic training. Most participants exercised more than twice a week and approximately half undertook some form of resistance training, but with only one third undertaking resistance training at least twice a week in line with current guidelines.

### 4.1 Participant characteristics

The reported mean age, male:female ratio and BMI characteristics were comparable to athletes attending the 1997 USA Transplant Games and the 2011 World Transplant Games (14, 24). The male:female ratio of ∼60:40 is reflective of the broader transplant community where, although females account for a greater proportion of transplant donations (50%–66%), the greater proportion of recipients are male (62%–65%) (25, 26). The greater age of the competitors in this current study when compared to non-transplant athletes [21–22 years of age; (27)] can, in part, be accounted for by our participant inclusion criteria of 18+ years, participants competing according to age (i.e., 18–29, 30–39, 40–49, 50–59, 60–69, 70+ years) and the mean age at transplant being largely in the mid 40's (25). While the body mass of competitors is comparable to non-transplantees (27), BMI was greater, potentially due to altered body composition subsequent to periods of ill health prior to transplant, an older competitive population or medication side effects (11). Most competitors (71%) reported being involved in exercise training to some degree prior to their transplant, which is similar to Johnson et al. (81%) for US athletes at the 2011 WTG (24), as were the breakdown of competitors considered as national/international (∼25%) and recreational standard (∼50%). It is therefore reasonable to surmise the general characteristics of competitive organ-recipients are likely to be older, predominantly male, with an increased BMI than their non-transplant counterparts, with a sizeable proportion having some history of exercise participation before transplant.

### 4.2 Sport and event participation

The most frequently participated sports within the current study populations were court-based sports followed by track athletics, field athletics, swimming and cycling. Johnson et al. (24), explored event participation of 248 transplantees competing in the 2011 WTG in Gothenburg, where the frequency of track, swimming and cycling events were similar to our data (representing 27%, 15% and 10% of participants, respectively). Conversely, court-based sports were not reported within the top five predominant sports (i.e., track and field, swimming, cycling, golf and bowling) and field events were combined with track events. It is possible that the combination of both BTG and WTG participants, recruitment procedures, national representation/event selection, and criteria for sport participation may have contributed to differences between studies with respect to court-based competitor numbers. However, the current study furthers the data from Johnson et al., (24) by providing a specific breakdown of the number of sports and sport specific events competitive organ-recipients participate in. Within Transplant Games, competitors can compete in up to five sporting disciplines (3). Our data shows most competitors undertook multiple events within their predominant sport and, in general, one other sporting discipline. Where the physiological demands are lower (e.g., LPD sports; where participants tended to be older, heavier, and have greater BMI) or less events are available, a greater variety of sports may be pursued. Such sporting behaviours have likely evolved from the ethos of Transplant Sport, which encourages participation for enjoyment across a variety of sports rather than specialisation and performance *per se* (1).

### 4.3 Training frequency, intensity and duration

When considering the general population, it is recommended that adults undertake 150 min of moderate physical activity (e.g., minimum of 30 min per session over 4–5 days; TL = 600–750 AU) or 60–75 min of vigorous activity each week (TL = 480–600 AU) along with at least two sessions of strength training [NICE guidelines, (28)]. Based on the data for the whole group, most competitors did appear to be undertaking sufficient exercise sessions, i.e., 80% undertaking three or more sessions each week, but at a low to moderate intensity (i.e., RPE of 4–5, equivalent to ∼13–14 on 6–20 Borg scale (29). It is possible that a combination of unsupervised training and physiological limitations to exercise likely contribute to the overall lower intensity of training sessions reported. Indeed, Hames et al. (11) reported that 53% of competitive organ-recipients perceived there were limitations preventing them from performing at their potential, just 29% perceived they trained equally to non-transplant competitors, 16% reduced their training session intensity once started, and 45% considered they did not recover as well as non-organ recipient competitors. Nevertheless, sport specific training sessions were generally undertaken at greater intensities (RPE = 5) with a graded RPE response to exercise duration, (i.e., greater RPE for shorter duration sessions). Therefore, participants appear to be able to discriminate exercise training intensities appropriately, despite the daily use of numerous medications and potential exercise limitations. However, only half of the participants undertook resistance training, with just one third undertaking resistance training twice or more each week. Future work should therefore consider how to increase the uptake and awareness of resistance training in the competitive transplant population.

### 4.4 Training load

#### 4.4.1 Total training load

The mean weekly TL across all competitors was 2,260 AU, and 2,762 AU for those competitors who actively trained for a predominant sport. These values are greater than reported for elite non-transplant athletes where the same method of quantifying training load has been used (30–33). When considered in relation to specific sports, lower weekly training loads for non-transplant athletes have been reported for national (30) and international middle-distance track athletes (33), field athletes (30) and volleyball players (31) (i.e., court-based sports). Training loads for cyclists, however, were similar to the current study (32), whereas training loads for national standard swimmers (34) were greater than the current study. One reason for the lower TL in swimmers in our study is potentially the lower number of swimming specific sessions when compared to non-transplant swimmers, undertaking more than 13 sessions per week (34). Interestingly, the competitive cyclists in the current study had the most comparable TL to non-transplant competitors and undertook the least number of non-sport specific events.

#### 4.4.2 British compared to World Transplant Games competitors

Participants competing at either the BTG or WTG similarly reported exercising more than twice a week. However, the total TL for those competing at the WTG was greater due to a greater frequency and intensity of training, and tended to be distributed among the greater training load categories. Such a difference in TL likely reflects the varied reasons for competing in relation to competitive standard, whether to win medals or for more social aspects of the Games (11). Nevertheless, most competitors in the current study were undertaking an acceptable level of physical activity with wide ranging reasons for participating, but most likely gaining from the recognised health and quality of life benefits of increased physical activity. Furthermore, our data demonstrates that a wide range of activities and intensities can be undertaken by this population, which may encourage less active individuals to try a wider spectrum of exercise modes for eliciting potential health gains.

#### 4.4.3 Transplant type

Although there were no overall differences in TL between transplant type, effect size values indicated meaningful differences did exist. Indeed, these effects concur with established physiological differences in exercise capacity following organ transplant, namely that thoracic organ-recipients (i.e., heart, lung) have greater impairments than kidney and liver recipients (35). Furthermore, pre-transplant limitations for heart and lung recipients relate to the heart and lungs *per se*, whereas kidney and liver recipients limiting factors relate to more ‘indirect’ effects of severe chronic disease. However, most organ-recipients continue to demonstrate such limiting effects post-transplant, with all demonstrating limitations to peripheral skeletal muscle function (35). Our data therefore suggests that established limitations to exercise capacity in relation to the general organ-recipient population are evident in the training loads undertaken by competitors at national and international events.

### 4.5 Training modality

The most common training modality reported across sports was resistance training, with 43% of all competitors undertaking this type of training. Those sports reporting the greatest involvement in resistance training each week were track athletics, field athletics and cycling. Similarly, gym based aerobic training was undertaken by 30% of all athletes, with the same three sports represented to similar extents. Both resistance training and aerobic exercise are encouraged by physicians and transplant units often within one-year post-transplant (8, 36, 37), which may help to explain their greater level of engagement. However, it should also be acknowledged that involvement in resistance training was still less than half of the participants and, as such, most participants did not follow conventional exercise guidelines regarding resistance training. Similarly, although gym-based aerobic exercise was undertaken frequently in most sports, it was not undertaken to a large extent with regard to actual TL. The only type of training modality undertaken across sports to an equal or greater frequency was sport specific training (i.e., track in track athletes, cycling in cyclists etc.). Indeed, the greatest TL within each modality was generally sport specific training, being of a magnitude between 1,500–1900 AU, and generally performed at greater intensities. There were though some notable exceptions where the greatest sport specific TL was undertaken by cyclists (∼2,800 AU) and resistance training was the greatest TL undertaken by field athletes (∼1,900 AU) compared to all other sports (∼726–1,015 AU). For most competitors, a TL of similar magnitude to specific training was also observed (i.e., ∼1,500 AU) for nonspecific modes, likely reflecting supplementary training for the competitors secondary sporting discipline. Training modalities therefore represent a range of core modes with more focused training for a competitor's primary and secondary sports.

### 4.6 Determination of training load

The TL in the current study was determined by the product of RPE and session duration, as such, a potential contributor to TL variation may be the participants understanding of perception of effort. For example, if session RPE was perceived to be greater than it truly was, the result would be increased TL values. Although guidance was provided within the questionnaire regarding the use of the RPE scale it is unknown whether the participants had previous experience of interpreting the scale for training purposes or from laboratory testing. However, a difference of two RPE units (e.g., from RPE 6–8 for a 60 min session represents 360–480 AU, respectively) would result in a ∼30% greater training load calculation of ∼120 AU, and well within the reported standard deviation for most sporting disciplines in this study. Furthermore, as noted above, based on the exercise intensity (RPE) data, the current participants appear to be able to discriminate between sessions of different intensity. Error from the use of absolute RPE may therefore not be a major contributor to TL variation and, as noted above, may more simply be due to the wider range of training components undertaken by many competitors. Of greater consideration for perception of effort may be the effects of medication following recovery from transplant surgery and their potential physiological effects. Although glucocorticoid-induced atrophy and its clinical implications have been reviewed (38) and previous work has reported the wide range of medications routinely taken by competitive organ-recipients, any specific effects of these medications on exercise responses are generally undereported (11). One study of otherwise healthy men administered beta-blockade medication resulted in lower heart rate and oxygen consumption during exercise, although the RPE-exercise intensity relationship was unchanged (39). Whether such medications alter this relationship in competitive organ-recipients is unknown. Future research should consider RPE responses in relation to exercise intensity markers such heart rate, oxygen consumption and blood lactate concentration in competitive organ-recipients, in part to validate TL calculations, but more generally to allow accurate determination of exertion and exercise intensity in a population with wide ranging medications and potential effort mediators.

### 4.7 Application for coaches

The wide range of TL reported in the current study may be related to a similarly wide range in age (4, 14), reasons for participation and lack of specific training guidance; indeed, only 4% of competitive organ recipients reported having a coach (11). The value of coaching is undoubted, enabling an athlete to train and perform at their optimum by considering the specificity of training, training stimulus and the magnitude of training (40). However, many competitive organ-recipients would be categorized as Masters athletes (i.e., >35 years) and coaches may not be prepared for the nuances and requirements of such a population (41). Although we are unaware of any statistics regarding the number of masters athletes who use a coach, it is clear from the literature that having a coach as a Masters athlete is associated with greater intrinsic motivation relating to both pleasure and satisfaction with sporting activities (42). In addition to accepted age-related changes in physiology, coaches of competitive organ-recipients will also have to navigate the effect of chronic medication, physiological limitations, altered recovery, differences in exercise motivations and performance expectations to name but few. Avoiding training errors from imbalances between training stimulus and recovery, especially where athletes compete in multiple events and disciplines (43), also need to be considered. Consequently, there is a clear lack of sport specific training advice and guidance for competitive organ-recipients. A lack of knowledge regarding support of competitive organ-recipients has recently been emphasised from reviewing the knowledge base and beliefs of therapists supporting competitors attending Transplant Games (44). Due to a paucity of evidence-based research, guidance informing how therapists manage such competitors was drawn predominantly from other medical practitioner's experience. The consequence of which meant therapists perceived competitive organ-recipients as a vulnerable population reverting to a cautious approach to their management. Subsequently, there is a clear lack of research-based knowledge to inform coaching and therapeutic support in the management of competitive organ-recipients aiming to achieve optimal sporting performance.

### 4.8 Limitations

While the present study acknowledges that TL data collected retrospectively may result in potential recall error, the authors recognise there was no feasible alternative for obtaining typical training characteristics. However, without this data, collected in this manner, researchers would have no solid foundation with which to build effective training programmes or understand TL in competitive organ-recipients. Ensuring the method for quantification of TL was easy for participants to interpret when compiling their data (i.e., the 0–10 RPE scale and the exercise duration) was therefore of key importance. Several researchers have explored monitoring the intensity of training sessions in soccer players, throwers and runners using the 0–10 RPE scale and concluded that RPE can be considered a reliable method for monitoring TL (19, 45). Furthermore, Cejuela and Esteve-Lanao (46) reported that RPE methods (i.e., Borg 0–10 or 6–20 scales) were positively correlated with other approaches such as heart rate zones and training impulse (TRIMP). However, if RPE values were not taken within 30 min post session correlation discrepancies have been noted to potentially overestimate TL for low-intensity interval training and underestimate training loads for high intensity interval training (18). Therefore, error may have been introduced as competitors may lose time and intensity perspectives of the session (46). However, we are confident that as training components were logged relating to the training currently being undertaken, any error would be minimized.

A second limitation is that training data was collected for a “typical” in season training week corresponding to the time of completion of the questionnaire. However, this approach enabled a consistent period for TL comparison, ecological validity and real-world application. Future research should monitor each competitor's training over a typical 12-month macrocycle covering both on and off-season loads. Including other training aspects such as specific types of resistance training, balance, agility, coordination and flexibility will be informative for training developments. In addition, affecting factors such as alterations to medication or health status should be documented. Thirdly, our study focused upon those organ recipients who are regularly training and competing and could thus be considered as those with positive exercise (and likely health) experiences. Therefore, there is a limitation to the application of our findings to those organ recipients who are already active. Indeed, with approximately 1,500 participants at the 2017 World Games and 465 at the 2017 BTG (personal communication) our sample of *n* = 220 represents ∼11% of the competitor population. Expanding our knowledge of exercise barriers and behaviour's across both active and non-active organ recipients will improve our understanding of their impact upon health and quality of life markers.

Finally, although we have previously reported more general complications experienced by competitive organ-recipients (11), our study did not explore injuries or adverse events specifically during training or competing, or aspects relating to training and competing in the heat, high altitude, air pollution or the use of performance-enhancing strategies as outlined by Stylemans et al. (10). Such a wide range of factors potentially affecting performance align to those considered for non-transplantee competitors and form future research directions.

## 5 Conclusion

This study is the first to evaluate the training characteristics of competitive organ-recipients at both national and international Transplant Games, providing a critically important foundation to take training prescription for transplant sport forwards. Competitors regularly compete in multiple events within their main sport and often within a secondary sport. As a result, competitors TL reflect the ethos of transplant sport being subsequently diverse, with large variation and of a magnitude often greater than for non-transplant athletes competing in similar disciplines. There is, therefore, a clear need to increase our knowledge of training and TL components post-transplant across medical staff, rehabilitation practitioners, coaches and competitors alike. In doing so, this will enable coaches and therapists to support those competitors seeking to achieve optimal performance goals as well as supporting those organ-recipients with no prior training experience aiming at attaining minimal recommendations for physical activity and associated health benefits.

## Data Availability

The datasets presented in this article are not readily available because of potential identifiers within (anonymised) categorising variables. Requests to access the datasets should be directed to Thomas Hames, ab8077@coventry.ac.uk.
