# Supplementary material for: Training loads and practices of competitive organ-recipients at the British and World Transplant Games
Source: Front Sports Act Living. 2024 Nov 29;6:1445491. doi: 10.3389/fspor.2024.1445491 (PMC11637886; doi:10.3389/fspor.2024.1445491)
Supplement: Supplementary file 1 [file Table1.docx]

Supplementary Material

**Training loads and practices of competitive organ-recipients at the British and World Transplant Games.**

**Thomas Hames, Sheila Leddington-Wright, C. Douglas Thake, Stefan De Smet, Mike Price***

*** Correspondence:** Corresponding Author: mike.price@coventry.ac.uk

**Supplementary Table 1.** Frequency of transplant type within each main sporting discipline.

|  | Total | Heart | Kidney | Liver | Lung | Stem cell | Pancreas | Heart and Lung | Kidney and pancreas |
| --- | --- | --- | --- | --- | --- | --- | --- | --- | --- |
| Total n (%) | 218 | 35 (16%) | 95  (44%) | 48 (22%) | 11  (5%) | 21 (10%) | 1  (<1%) | 4  (2%) | 3  (1%) |
| Track | 51 | 10 (20%) | 16  (31%) | 14 (27%) | 2  (4%) | 7  (14) | - | 1  (2%) | 1  (2%) |
| Field | 30 | 8  (27%) | 9  (30%) | 4  (13%) | 2  (7%) | 5  (17%) | - | 1  (3%) | 1  (3%) |
| Cycling | 16 | 7  (44%) | 7  (44%) | - | - | 1  (6%) | 1  (6%) | - | - |
| Swimming | 26 | 2  (8%) | 12  (46%) | 9  (35%) | 2  (8%) | 1  (4%) | - | - | - |
| Court-based | 70 | 5  (7%) | 37  (53%) | 17 (24%) | 5  (7%) | 5  (7%) | - | 1  (1%) | - |
| HPD | 6 | - | 3  (50%) | 1  (17%) | - | 1  (17%) | - | 1  (17%) | - |
| LPH | 19 | 3  (16%) | 11  (58%) | 3  (16%) | - | 1  (5%) | - | - | 1  (5%) |

NB: N=3 participants excluded as they did not identify a main sport.

**Supplementary Table 2.** Training characteristics of TxA’s for a representative in-season training week. Specific sessions for each discipline are highlighted. RPE = Rating of Perceived Exertion, Min = minutes, Frq = Training frequency - number of training sessions per wk, N = number of athletes, TL = weekly training load.

| **Number of Athletes** | **All Athletes** | | | | | **Track Athletes** | | | | | **Field Athletes** | | | | | **Cycling Athletes** | | | | |
| --- | --- | --- | --- | --- | --- | --- | --- | --- | --- | --- | --- | --- | --- | --- | --- | --- | --- | --- | --- | --- |
|  | **n =218** | | | | | **n =51** | | | | | **n =30** | | | | | **n =16** | | | | |
| **Session type** | RPE | Min | Frq | N | TL | RPE | Min | Frq | N | TL | RPE | Min | Frq | N | TL | RPE | Min | Frq | N | TL |
| **Resistance training (Gym)** | *4* | *90* | *3* | *52* | *1096 +1758* | 5 | 45 | 2 | 24 | 822 +986 | 6 | 75 | 4 | 21 | 1904 +3190 | 6 | 75 | 2 | 7 | 874 +926 |
| **Aerobic session (Gym)** | *4* | *75* | *3* | *34* | *886 +791* | 5 | 60 | 3 | 15 | 1151 +1024 | 5 | 45 | 3 | 14 | 927 +898 | 6 | 60 | 2 | 5 | 852 +856 |
| **Aerobic classes (Gym)** | *5* | *75* | *2* | *11* | *783 +783* | 3 | 90 | 3 | 5 | 978 +1280 | 1 | 75 | 1 | 3 | 540 +393 | 8 | 60 | 3 | 3 | 1345 +474 |
| **Sprint distance track sessions** | *4* | *90* | *2* | *22* | *1003 +874* | 7 | 75 | 2 | 14 | 1224 +1035 | 6 | 60 | 2 | 6 | 675 +606 | 4 | 75 | 2 | 2 | 450 +212 |
| **Middle distance track sessions** | *4* | *90* | *2* | *18* | *904 +1195* | 7 | 75 | 2 | 14 | 1258 +1580 | 5 | 45 | 2 | 4 | 356 +236 | - | - | - | - | - |
| **Long distance track sessions** | *4* | *90* | *3* | *15* | *731 +440* | 6 | 60 | 2 | 14 | 768 +456 | 7 | 45 | 1 | 1 | 315 | - | - | - | - | - |
| **Jump sessions** | *4* | *90.* | *3* | *5* | *1273 +1213* | 5 | 45 | 1 | 2 | 195 +106 | 5 | 60 | 3 | 3 | 980 +900 | - | - | - | - | - |
| **Throwing sessions** | *4* | *75* | *3* | *20* | *1325 +3091* | 3 | 90 | 1 | 2 | 248 +159 | 5 | 60 | 3 | 18 | 1658 +3655 | - | - | - | - | - |
| **Sprint distance cycling** | *3* | *105* | *2* | *10* | *686 +523* | 7 | 60 | 2 | 3 | 720 +668 | - | - | - | - | - | 8 | 60 | 2 | 7 | 741 +532 |
| **Middle distance cycling** | *5* | *75* | *3* | *19* | *1182 +1516* | 4 | 90 | 2 | 5 | 726 +664 | - | - | - | - | - | 6 | 90 | 3 | 14 | 1782 +1897 |
| **Long distance cycling** | *8* | *75* | *2* | *9* | *1102 +1127* | 5 | 120 | 1 | 3 | 725 +439 | - | - | - | - | - | 5 | 150 | 2 | 6 | 1800 +1371 |
| **Sprint distance swimming** | *4* | *90* | *3* | *7* | *797 +542* | 6 | 30 | 1 | 1 | 180 | 6 | 60 | 3 | 4 | 874 +741 | 6 | 75 | 3 | 2 | 1163 +1008 |
| **Middle distance swimming** | *4* | *75* | *2* | *7* | *536 +539* | 4 | 75 | 1 | 5 | 294 +258 | 3 | 45 | 2 | 1 | 270 | 6 | 75 | 1 | 1 | 450 |
| **Long distance swimming** | *4* | *60* | *2* | *1* | *429 +285* | 3 | 60 | 1 | 1 | 180 | - | - | - | - | - | - | - | - | - | - |
| **Court-based sessions** | *5* | *90* | *3* | *8* | *1512 +2324* | 4 | 75 | 5 | 1 | 1500 | 4 | 45 | 1 | 4 | 150 +77 | 4 | 90 | 1 | 3 | 470 +321 |
| **Other** | *5* | *75* | *3* | *19* | *720 +720* | 5 | 60 | 3 | 12 | 820 +852 | 4 | 60 | 3 | 3 | 530 +165 | 7 | 30 | 4 | 4 | 465 +209 |
| **Athlete cumulative load** | *2762 +3583* | | | | | 2547 +2664 | | | | | 3671 +6189 | | | | | 4383 +4005 | | | | |
|  |  |  |  |  |  |  |  |  |  |  |  |  |  |  |  |  |  |  |  |  |
|  |  |  |  |  |  |  |  |  |  |  |  |  |  |  |  |  |  |  |  |  |
|  |  |  |  |  |  |  |  |  |  |  |  |  |  |  |  |  |  |  |  |  |
|  |  |  |  |  |  |  |  |  |  |  |  |  |  |  |  |  |  |  |  |  |
|  |  |  |  |  |  |  |  |  |  |  |  |  |  |  |  |  |  |  |  |  |
| **Number of Athletes** | **Swimming Athletes** | | | | | **Court-based Athletes** | | | | | **High physiological demand sports (HPD)** | | | | | **Low physiological demand sports (LPD)** | | | | |
|  | **n =26** | | | | | **n =70** | | | | | **n =6** | | | | | **n =19** | | | | |
| **Session type** | RPE | Min | Frq | N | TL | RPE | Min | Frq | N | TL | RPE | Min | Frq | N | TL | RPE | Min | Frq | N | TL |
| **Resistance training (Gym)** | 6 | 60 | 2 | 9 | 726 +581 | 5 | 60 | 3 | 25 | 1015 +1116 | 4 | 60 | 2 | 2 | 623 +711 | 5 | 45 | 2 | 5 | 564 +407 |
| **Aerobic session (Gym)** | 6 | 60 | 3 | 4 | 855 +687 | 6 | 60 | 3 | 21 | 814 +642 | 5 | 60 | 2 | 4 | 510 +369 | 4 | 45 | 2 | 2 | 390 +297 |
| **Aerobic classes (Gym)** | 2 | 60 | 1 | 1 | 90 | 5 | 60 | 2 | 6 | 578 +423 | - | - | - | - | - | - | - | - | - | - |
| **Sprint distance track sessions** | 6 | 105 | 2 | 1 | 1260 | 5 | 45 | 3 | 4 | 791 +874 | 5 | 75 | 3 | 1 | 1575 | - | - | - | - | - |
| **Middle distance track sessions** | 8 | 60 | 2 | 2 | 638 +456 | 5 | 60 | 3 | 4 | 780 +159 | 7 | 60 | 1 | 2 | 368 +329 | 8 | 60 | 1 | 1 | 480 |
| **Long distance track sessions** | - | - | - | - | - | 4 | 105 | 4 | 1 | 630 | - | - | - | - | - | - | - | - | - | - |
| **Jump sessions** | - | - | - | - | - | 10 | 30 | 4 | 1 | 2700 | 8 | 90 | 3 | 1 | 2880 | - | - | - | - | - |
| **Throwing sessions** | 3 | 15 | 1 | 1 | 45 | 3 | 60 | 5 | 3 | 1125 +1366 | 3 | 45 | 2 | 1 | 270 | 7 | 60 | 1 | 1 | 420 |
| **Sprint distance cycling** | - | - | - | - | - | 9 | 45 | 5 | 1 | 810 | 5 | 30 | 2 | 1 | 75 | - | - | - | - | - |
| **Middle distance cycling** | 6 | 45 | 1 | 2 | 225 +21 | 7 | 45 | 3 | 3 | 500 +308 | 4 | 60 | 2 | 2 | 330 +212 | 6 | 60 | 2 | 1 | 720 |
| **Long distance cycling** | - | - | - | - | - | 6 | 90 | 4 | 1 | 540 | 3 | 75 | 1 | 2 | 203 +32 | - | - | - | - | - |
| **Sprint distance swimming** | 7 | 75 | 2 | 16 | 848 +465 | 3 | 45 | 4 | 2 | 420 +424 | 4 | 45 | 2 | 1 | 360 | - | - | - | - | - |
| **Middle distance swimming** | 6 | 60 | 2 | 15 | 780 +652 | 4 | 45 | 2 | 4 | 199 +110 | 4 | 45 | 2 | 5 | 387 +402 | - | - | - | - | - |
| **Long distance swimming** | 5 | 75 | 2 | 6 | 597 +220 | 2 | 30 | 5 | 1 | 120 | 2 | 75 | 1 | 1 | 150 | - | - | - | - | - |
| **Court-based sessions** | 5 | 60 | 2 | 2 | 390 +212 | 6 | 90 | 3 | 50 | 1863 +2584 | 6 | 15 | 1 | 1 | 90 | 4 | 75 | - | 6 | 630 +784 |
| **Other** | 4 | 75 | 1 | 2 | 248 +159 | 6 | 60 | 3 | 6 | 905 +1009 | 5 | 75 | 3 | 3 | 1050 +1074 | 3 | 120 | 2 | 6 | 564 +336 |
| **Athlete cumulative load** | 2051 +1047 | | | | | 2645 +3308 | | | | | 2595 +2247 | | | | | 1182 +801 | | | | |
