# Supplementary material for: Training loads and practices of competitive organ-recipients at the British and World Transplant Games
Source: Front Sports Act Living. 2024 Nov 29;6:1445491. doi: 10.3389/fspor.2024.1445491 (PMC11637886; doi:10.3389/fspor.2024.1445491)
Supplement: Supplementary file 2 [file Datasheet2.pdf]

The Competitive Organ Transplant Athlete

Participant Information and Consent

PARTICIPANT INFORMATION SHEET

POST GRADUATE STUDENT PROJECT

SCHOOL OF LIFE SCIENCES

Thank you for considering helping one of our students with their research work. This form explains what you will be asked to do. If you have any questions about this, please ask the student.

By signing this form, you agree to take part in the study. However, please note that you are free to stop taking part at any time.

Information about the project/Purpose of the project

This project investigates the athletic demographic of the competitive organ transplant recipient.

Why have I been chosen?

We are approaching all athletes 18+ participating at the World and British Transplant Games to see if they would be willing to take part in an online survey to investigate the sporting history of the organ transplant athlete.

Do I have to take part?

You do not have to take part in this research project if you do not want to and you do not need to give any reason if you decide not to take part.

What do I have to do?

By choosing to participate in this project, you will be asked to complete an online questionnaire surveying the athletes participating in the World and British Transplant Games.

1. You will be asked to provide some personal information on your gender, age, nationality, ethnicity, job and education.

2. You will then be asked some background information on your transplant including reason for transplant, duration since transplant and medications.

3. The survey will then ask for information on physical activity levels and sporting background, and current sporting interest.

4. The main section of the survey asks questions relating to a 'normal training week', asking you to provide information on days per week you train, rest, types of training, intensity of training.

5. The final questions will ask you to provide information on how you recover between training sessions, and your beliefs or barriers to improving your performance.

What are the risks associated with this project?

Potential information could be visible when filling out this form in a public place, therefore a link to the survey will also be supplied so the survey can be completed at home or in a place of privacy.

What are the benefits of taking part?

By participating in this survey, your information will allow us to build an understanding of the range of training undertaken of transplant athletes, and associated perceptions. In the long term, this will enable future research to identify target areas for training interventions and management alongside developing transplantee specific athlete and local education.

Withdrawal options

You are free to stop taking part in this study at any time and you do not have to give any reason for this.

Once you are happy and have COMPLETED the survey only then is information irretrievable as answers are pooled together. However, answers cannot be linked to individuals, thus keeping the survey anonymous.

Data protection & confidentiality

Your consent to participate in this study will be confidential. Once you have given your consent, this form will be stored securely and appropriately by which only the researcher and supervisor has access to. The consent forms will not be stored with other data that belongs to the study, therefore ensuring no connections. These will be held for the maximum period of 5 years, and then will be destroyed securely. This time frame is determined by the need for access to this information in the unfortunate case of an unanticipated problem or a complaint. At the expiry date this information will be destroyed securely by the Faculty Research Support Officer.

Throughout this investigation, all information is kept anonymous and blind to the researcher. Participants codes are used with the electronic documents so that individuals cannot be identified.

Who should you talk to if you have questions or you wish to make a complaint?

If you have any questions or queries Thomas Hames will be happy to answer them. If they cannot help you, you can speak to Dr Mike Price.

If you have any questions about your rights as a participant or feel you have been placed at risk you can contact Dr Mike Price.

What will happen with the results of the study?

Any data/ results from your participation in the study will be used by Thomas Hames as part of their project work. The data will also be available to Dr Mike Price as director of studies and the supervisory team of Dr Doug Thake and Sheila Leddington Wright. It may also be published in scientific works, but your name or identity will not be revealed.

Who has reviewed this study?

This study has ethical approval from Coventry University

Key contact details

[redacted]

Please tick YES below to confirm that by filling in this questionnaire you are confirming that: You give consent to use your questionnaire answers in this research study. You have read and understood the information above about the study You understand that your participation is voluntary and that you are free to withdraw at any time without giving a reason You understand that all the information you provide is anonymous If you do not agree then please tick NO to exit the questionnaire.

YES

NO

About you

This section asks you to provide a range of personal information.

Gender:

Male Female

Age:

Height (cm):

Weight (kg):

Nationality:

Afghan Albanian Algerian

Andorran Angolan Argentinian

Armenian Australian Austrian

Azerbaijani Bahamian Bangladeshi

Barbadian Belorussian Belgian

Beninese Bhutanese Bolivian

Bosnian Brazilian Bruneian

Bulgarian Burmese Burundian

Cambodian Cameroonian Canadian

Chadian Chilean Chinese

Colombian Congolese Croatian

Cuban Cypriot Czech

Danish Dominican Ecuadorean

Egyptian Salvadorean English

Eritrean Estonian Ethiopian

Fijian Finn French

Gabonese Gambian Georgian

German Ghanaian Greek

Grenadian Guatemalan Guinean

Guyanese Haitian Dutchman

Honduran Hungarian Icelander

Indian Indonesian Iranian

Iraqi Irish Israeli

Italian Jamaican Japanese

Jordanian Kazakh Kenyan

Korean Kuwaiti Laotian

Latvian Lebanese Liberian

Libyan Liechtensteiner Lithuanian

Luxembourger Macedonian Madagascan

Malawian Malaysian Maldivian

Malian Maltese Mauritanian

Mauritian Mexican Moldovan

Monacan Mongolian Montenegrin

Moroccan Mozambican Namibian

Nepalese Nicaraguan Nigerien

Nigerian Norwegian Pakistani

Panamanian Paraguayan Peruvian

Filipino Pole Portuguese

Qatari Romanian Russian

Rwandan Saudi Scottish

Senegalese Serbian Singaporean

Slovak Slovenian Somali

Spaniard SriLankan Sudanese

Surinamese Swazi Swede

Swiss Syrian Taiwanese

Tadzhik Tanzanian Thai

Togolese Trinidadian Tunisian

Turk Ugandan Ukrainian

American Uruguayan Uzbek

Venezuelan Vietnamese Welsh

Yemeni Yugoslav Zambian

Zimbabwean Prefer not to answer

Ethnic origin:

White

White - Scottish Irish Traveller

Gypsy or Traveller

Other White background

Black or Black British - Caribbean

Black or Black British - African

Other Black background

Asian or Asian British - Indian

Asian or Asian British - Pakistani

Asian or Asian British - Bangladeshi

Chinese

Other Asian background

Mixed - White and Black Caribbean

Mixed - White and Black African

Mixed - White and Asian

Other mixed background

Arab

Other ethnic background

Not known

Prefer not to answer

Post code:

How long have you lived at this post code:

Dates need to be in the format 'DD/MM/YYYY', for example 27/03/1980.

Education - Highest current academic qualification:

What is your current occupational status?:

Employed

Unemployed

Self employed

Student Retired

Is your occupation a FULL time or PART time post?:

Full time

Part time

About your transplant

1st Transplant type:

The following section asks you about your transplant.

Heart

Kidney

Liver

Lung

Pancreas

Intestine

Thymus

Marrow

Other

If you selected Other, please specify:

What was the reason for the transplant?:

How long did it take between initial diagnosis for condition to receiving the 1st transplant?:

Your age at 1st transplant?:

Approximate date of 1 organ transplant?:

Dates need to be in the format 'DD/MM/YYYY', for example 27/03/1980.

(dd/mm/yyyy)

Further organ transplantations, reason and age at the time of each transplantation?

Yes

No

Have you had any complications or rejection episodes post-transplant?:

If yes, how what was the complication, and how long ago was this?

Age of donor transplants (if known)?:

Your medications

The following section explores the medications currently used to manage your transplants and other pathologies while you are competing.

List any medication you are currently taking:

	Medication	Dose	Times per day
Medication 1:			
Medication 2:			
Medication 3:			
Medication 4:			
Medication 5:			
Medication 6:			
Medication 7:			
Medication 8:			
Medication 9:			
Medication 10:			

Your sporting background

The following section explores your participation in sport pre-transplantation.

Did you participate in sport before your transplant?:

Yes

No

At what level did you previously compete?: Name sporting event(s) and whether it was International, National, County or Club level and highest national rank if known?

For a regular training week, how many days per week did you train:

0 1 2 3 4 5 6 7

For a regular training week, how many sessions per week were you participating in?:

Please don't select more than 1 answer(s) per row.

	0	1	2	3	4	5	6	7	8	9	10
Gym based training session											
Athletics track based training session											
Field based training session (Jumps / throws)											
Swimming training session											
Cycling training session											
Court based training session											
Other											

For a regular training week, how long would the average training session last.

Please don't select more than 1 answer(s) per row.

	0	15 Min	30 min	45 min	60 min	75 min	90 min	105 min	120 min	135 min	150 min
Gym based											

training session											
Athletics track based training session											
Field based training session (Jumps / throws)											
Swimming training session											
Cycling training session											
Court based training session											
Other											

The following question uses the Rate of Perceived Exertion scale (RPE) establish how physically demanding you felt each of the sessions were (Borg 1982).

RPE SCALE - HOW INTENSE DOES IT FEEL?

1 = Very easy

2 = Easy

3 = Moderate

4 = Somewhat hard

5 = Hard

6

7 = Very hard

8

9

10 = Maximal

For a regular training week, how intense were the training sessions you participated in. Please tick the average intensity level best related to each

Please don't select more than 1 answer(s) per row

	N/A - Not applicable	0 - Rest	1 - Very easy	2 - Easy	3 - Moderate	4 - Somewhat hard	5 - Hard	6 -	7 - Very hard	8 -	9 -	10 - Maximal
Gym based training session												
Athletics track based training session												
Field based training session (Jumps / throws)												
Swimming training session												
Cycling training session												
Court based training session												
Other												

For how many years/months had you been training at this level?:

Your initial physical activity involvement post-transplant

This section explores your reintroduction to physical activity post-transplant

Did you receive any advice on exercise post-transplant?

No

Doctor

Therapist

Coach

Friend

Family

Other recipients

Gym instructor

Personal trainer

Other

What did they advise you to do?

How many months post-transplant did you start to participate in physical exercise (general or as part of rehabilitation)?:

What type/s of exercise were you participating in 12 months post-transplant. For a normal week how

many sessions were you participating in, and were they GUIDED (devised by another professional) or your own plan. Please don't select more than 1 answer(s) per row.

	0	1	2	3	4	5	6	7	8	9	10
Resistance training sessions											
Resistance training sessions (GUIDED)											
Gym based bike / treadmill aerobic sessions											
Gym based bike / treadmill aerobic sessions (GUIDED)											
Gym class sessions Gym class sessions (GUIDED)											
Running sessions											

Running sessions (GUIDED)											
Outdoor cycling											
Outdoor cycling (GUIDED)											
Swimming sessions											
Swimming sessions (GUIDED)											
Court based sessions											
Court based sessions (GUIDED)											
Other											

What was your top 3 reasons for wanting to start to compete?:

Your current sporting interests

This section will explore your current sporting aims, and your competition goals.

For how many years have you been participating in transplant sports?:

If you selected Other, please specify:

What is your current MAIN sporting event?:

Badminton

Basketball

Cycling - 5k

Cycling - 30k

Darts Golf

Kayak

Padel

Petaque

Squash

Swimming - 50m freestyle

Swimming - 100m freestyle

Swimming - 200m freestyle

Swimming - 400m freestyle

Swimming - 50m breastroke

Swimming - 100m breastroke

Swimming - 50m backstroke

Swimming - 100m backstroke

Swimming - 50m butterfly

Swimming - 200m Individual medley

Table Tennis

Ten Pin Bowling

Tennis

Track and Field - 100m

Track and Field - 200m

Track and Field - 400m

Track and Field - 800m

Track and Field - 1500m

Track and Field - 5000m / 3000m

Track and Field - Shot put

Track and Field - Ball Throw

Track and Field - Discus

Track and Field - Javelin

Track and Field - Long jump

Track and Field - High jump

Triathlon

Volleyball Other

If you selected Other, please specify:

Do you currently have a coach for your MAIN event?

Yes No

How long have you been coached towards your MAIN event?

How long have you been competing at your current MAIN event post-transplant?:

If you also compete in SECONDARY events, highlight which ones you currently compete in?:

Badminton

Badminton - Doubles Basketball

Cycling - 5k

Cycling - 30k

Cycling - 20k Team

Darts

Darts -Team

Golf

Golf - Team

Kayak

Padel

Petanque

Petanque - Doubles

Squash

Swimming - 50m Freestyle

Swimming - 100m Freestyle

Swimming - 200m Freestyle

Swimming - 400m Freestyle

Swimming - 50m Breastroke

Swimming - 100m Breastroke

Swimming - 50m Backstroke

Swimming - 100m Backstroke

Swimming - 50m Butterfly

Swimming - 200m Individual medley

Swimming - 4x50m Freestyle relay

Swimming - 4x50m Medley relay

Table tennis

Table tennis - Doubles

Ten pin bowling

Ten pin bowling - Doubles

Tennis

Tennis - Doubles

Track and Field - 100m

Track and Field - 200m

Track and Field - 400m

Track and Field - 800m

Track and Field - 1500m

Track and Field - 5000m / 3000m

Track and Field - 4x100m Relay

Track and Field - 4x400m Relay

Track and Field - Shot put

Track and Field - Ball throw

Track and Field - Discus

Track and Field - Javelin

Track and Field - Long jump

Track and Field - High jump Triathlon

Volleyball

Other

If you selected Other, please specify:

What is the highest level of competition you currently compete at?:

World Transplant Games

British Transplant Games

List your Personal Best (PB's) for your events. Only fill in the ones you compete at.

Cycling 5k

Cycling 30k

Cycling 20k Team

Golf

Kayak

Padel

Swimming - 50m Freestyle

Swimming - 100m Freestyle

Swimming - 200m Freestyle

Swimming - 400m Freestyle

Swimming - 50m Breastroke

Swimming - 100m Breastroke

Swimming - 50m Backstroke

Swimming - 100m Backstroke

Swimming - 50m Butterfly

Swimming - 200m Individual medley

Track and Field - 100m

Track and Field - 200m

Track and Field - 400m

Track and Field - 800m

Track and Field - 1500m

Track and Field - 5000m/3000m

Track and Field - Shot put

Track and Field - Ball throw

Track and Field - Discus

Track and Field - Javelin

Track and Field - Long jump

Track and Field - High jump

Triathlon

Other

Could you please select the main reasons you continue to participate in sport?:

For fun

To be part of the transplant community

To encourage a healthier lifestyle

To improve my fitness

To compete at new events

To compete at events I had done BEFORE the transplant

To win national events

To win international events

To break records

Other

If you selected Other, please specify:

Do you currently have support for your training from any of the following?:

Chiropractor

Nutritionist

Physiotherapist

Psychologist

Podiatrist

Sports Therapist

Strength and Conditioning coach

Other

If you selected Other, please specify:

Who provides you with the support?:

Chiropractor

Coach - Qualified

Doctor

Exercise Physiologist

Exercise Psychologist

Fellow athlete

Gym instructor

Nutritionist

Personal Trainer

Physiotherapist

Sports Scientist

Sports Therapist

Strength and Conditioning coach

Other

If you selected Other, please specify:

Your training and event preparation

This section reviews current strategies used to help athletes prepare for training sessions and event preparation.

Do you currently have a set protocol / warm up to help you prepare for COMPETITION? (Brief description, types of warm up and duration)

How long does your normal competition warm up last?

Do you currently have a set protocol / warm up to help you prepare for TRAINING sessions? (Brief description, types of warm up and duration)

How long does your normal training session warm up last?

Is there anything you feel in particular inhibits your event preparation? (E.g. difficulty increasing heart rate, quality of breathing etc)

If so, Is there anything you have done to try and improve on this?

Your training loads (Normal training week)

This section looks at the load and types of training you currently participate in.

When calculating session durations, please round to the nearest 15minutes, e.g: 1=15min, 2=30min, 3=45min, 4=60min, 5=75min, 6=90min,7=105min, 8=120min, 9=135min, 10=150min etc.

For a normal training week, how many days per week would you regularly train for your events? (all types of training sessions): 1 2 3 4 5 6 7

For a normal training week, how many training sessions would this involve? (Include multiple sessions within a day): 1 2 3 4 5 6 7 8 9 10 11 12 13 14 15

For a normal training week, what types of sessions would you participate in?

Crossfit

Cycling

Gym based cardiovascular training (Treadmill etc)

Gym based resistance training

Pilates

Road running

Self-directed

Track and Field

Yoga

Other

If you selected Other, please specify:

The following questions consider your training load using a scale of 0-10.

Only fill in the typs of sessions you participate in, leave blank and SKIP sessions you do not participate in.

In regards to session DURATION, round to the nearest 15 minutes: 1=15min, 2=30min, 3=45min, 4=60min, 5=75min, 6=90min, 7=105min, 8=120min, 9=135min, 10=150min.

In regards to the session INTENSITY question, refer to the Rate of Perceived Exertion scale (RPE) to judge on average how physically demanding sessions felt (Borg 1982).

RPE SCALE - HOW INTENSE DOES IT FEEL?

1 = Very easy21 / 31

2 = Easy

3 = Moderate

4 = Somewhat hard

5 = Hard

6

7 = Very Hard

8

9

10 = Maximal effort

If in a normal training week you participate in GYM based sessions, please can you identify what type, how many, the average duration and perceived intensity using the scales noted above. If you do not do any of these, please SKIP and move to the next.

Please don't select more than 1 answer(s) per row.

	0	1	2	3	4	5	6	7	8	9	10
Resistance training (Number of sessions)											
Resistance training (Average RPE)											
Resistance training (DURATION)											

Aerobic training (Number of Sessions)											
Aerobic training (Average RPE)											
Aerobic training (DURATION)											

Aerobic classes (Number of sessions)											
Aerobic classes (Average RPE)											
Aerobic classes (DURATION)											

If in a normal training week you participate in ATHLETICS TRACK based sessions, identify what type, how many, average duration and intensity using the scales noted above. If you do not do any of these, please SKIP and move to the next.

Please don't select more than 1 answer(s) per row.

	0	1	2	3	4	5	6	7	8	9	10
Sprint track sessions (Number of sessions)											
Sprint track sessions (Intensity RPE)											
Sprint track sessions (Average DURATION)											

Middle distance track sessions (Number of sessions)											
Middle distance track sessions (Intensity RPE)											
Middle distance track sessions (Average											

DURATION)											

Long distance track sessions (Number of sessions)											
Long distance track sessions (Intensity RPE)											
Long distance track sessions (Average DURATION)											

If in a normal training week you participate in FIELD (Jump/Throw) sessions, identify what type, how many, average duration and intensity using the scales noted above. If you do not do any of these, please SKIP and move to the next.

Please don't select more than 1 answer(s) per row.

	0	1	2	3	4	5	6	7	8	9	10
Jumping based field sessions (Number of sessions)											
Jumping based field sessions (Intensity RPE)											
Jumping based field sessions (Average DURATION)											

Throwing based field sessions (Number of sessions)											

Throwing based field sessions (Intensity RPE)											
Throwing based field sessions (Average DURATION)											

If in a normal training week you participate in CYCLING sessions, identify what type, how many, average duration and intensity using the scales noted above. If you do not do any of these, please SKIP and move to the next.

Please don't select more than 1 answer(s) per row.

	0	1	2	3	4	5	6	7	8	9	10
Sprint cycling sessions (Number of sessions)											
Sprint cycling sessions (Intensity RPE)											
Sprint cycling sessions (Average DURATION)											

Middle distance cycling sessions Number of sessions)											
Middle distance cycling sessions (Intensity RPE)											
Middle distance											

cycling sessions (Average DURATION)											

Long distance cycling sessions (Number of sessions)											
Long distance cycling sessions (Intensity RPE)											
Long distance cycling sessions (Average DURATION)											

If in a normal training week you participate in SWIMMING sessions, identify what type, how many, average duration and intensity using the scales noted above. If you do not do any of these, please SKIP and move to the next.

Please don't select more than 1 answer(s) per row.

	0	1	2	3	4	5	6	7	8	9	10
Sprint swimming sessions (Number of Sessions)											
Sprint swimming sessions (Intensity RPE)											
Sprint sessions swimming (Average DURATION)											

Middle distance											

swimming sessions Number of sessions)											
Middle distance swimming sessions (Intensity RPE)											
Middle distance swimming sessions (Average DURATION)											

Long distance swimming sessions (Number of sessions)											
Long distance swimming sessions (Intensity RPE)											
Long distance swimming sessions (Average DURATION)											

If in a normal training week you participate in COURT (Volleyball, tennis, table tennis etc) training sessions, identify what type, how many, average duration and intensity using the scales noted above. If you do not do any of these, please SKIP and move to the next.

Please don't select more than 1 answer(s) per row.

	0	1	2	3	4	5	6	7	8	9	10
Court based training session (Number of sessions)											
Court based											

training session (Intensity RPE)											
Court based training session (Average DURATION)											

If there are any other type of training you regularly participate in, please give details of: Type of session, Number of sessions, Intensity (RPE) and DURATION (multiples of 15min) in the box below.

Do you perceive there is anything currently limiting your ability to perform at your potential?

Yes No

If yes, what do you percieve is limiting your athletic ability?

Is there anything you are you currently doing to work on this?

Recovery

The following section explores any current recovery strategies you may have adopted to enhance your ability to compete or train.

At present, how long do you feel it takes for you to fully recover after a hard training session or competition?:

Do you currently follow any recovery protocol after competing or following training sessions?:

None at present

Gentle jog

Stretch (When feel tight)

Stretch (After sessions)

Foam roll (When feel tight)

Foam roll (After sessions)

Cryotherapy (Immersed)

Cryotherapy (Ice pack)

Structured plan

Other

If you selected Other, please specify:

If you use a recovery strategy, indicate which type/s of sessions you regularly use it after?

For a normal training week, please indicate the proportion of sessions you manage to carry out a recovery protocol after:

After every session

Only after competition

After a hard session (RPE 7+)

After 75% of sessions

After 50% of sessions

After 25% of sessions

Other

If you selected Other, please specify:

How long following a race or training session do you feel it takes for your heart rate (HR) to return to a resting state?:

Please don't select more than 1 answer(s) per row.

In relation to training on BACK to BACK DAYS, how recovered do you feel before starting the second day?

Please don't select more than 1 answer(s) per row.

	1 = VERY sore, tired stressed, poor recovery	2 = Sore more than normal, tired more than normal, restless	3 = Normal	4 = Feeling good	5 = VERY positive, fresh, relaxed, fully recovered
Fatigue					
Muscle soreness					
Stress level					

Mood					
Heart rate recovery					

In relation to back to back training sessions occurring on the SAME DAY, how recovered do you feel before starting the second session?

	1 = VERY sore, tired stressed, poor recovery	2 = Sore more than normal, tired more than normal, restless	3 = Normal	4 = Feeling good	5 = VERY positive, fresh, relaxed, fully recovered
Fatigue					
Muscle soreness					
Stress level					
Mood					
Heart rate recovery					

Compared to an event matched non-transplantee athlete, do you perceive you can train as the same session load for the same intensity?

Needs adjusting from the start

25% of the time

50% of the time

75% of the time

100% of the time

If not 100% of the time, what do you often change about your training session?

Same session - just longer breaks

Same session - just reduce intensity

Same Intensity - reduce reps / sets

Other

If you selected Other, please specify:

Compared to an event matched non-transplantee athlete, do you perceive you recover equally for back to back training sessions on the SAME DAY?:

No Yes

If not, what do you feel is affecting your ability to train at the same load?:

Compared to an event matched non-transplantee athlete, do you feel you recover equally for training sessions on BACK to BACK DAYS?:

No

Yes

If not, what do you feel is affecting your ability to train?:

End of Questionnaire

Thank you very much for your time. Your contribution will make a difference by helping to build a knowledge base to develop and support the competitive organ transplantee sporting demographic.
